# A Case of Persistent Low Back Pain in a Young Female Caused by a Trauma-Induced Schmorl’s Node in the Lumbar Spine Five Vertebra

**DOI:** 10.7759/cureus.1502

**Published:** 2017-07-21

**Authors:** Kurt M Mohty, Divneet Mandair, Brent Munroe, Deborah Baldemor

**Affiliations:** 1 College of Medicine, University of Arizona; 2 Department of Orthopaedic Surgery, University of Arizona; 3 Department of Family Medicine, University of Arizona

**Keywords:** low back pain, schmorl’s nodes, intervertebral disk

## Abstract

Physicians are often faced with managing difficult conditions such as chronic lower back pain. Intervertebral disk herniation typically occurs horizontally, leading to impingement of the spinal cord which can potentially cause radicular symptoms or other spinal cord pathologies; however, disk herniations can also occur vertically and extend through the endplate of an adjacent cranial or caudal vertebra: a phenomenon known as a Schmorl’s node. Although Schmorl’s nodes can be seen in many asymptomatic individuals, they can be a cause of degenerative disk disease and low back pain. An 18-year-old female with a history of trauma presented to urgent care with increasing lower back pain for the past six weeks. Four months prior, she was struck by a motor vehicle while riding her bicycle, and she had residual back pain since then. Plain radiography at the time of the accident showed no acute abnormalities. She had no other associated symptoms. On presentation, her vital signs were within normal limits, and her physical examination was largely unremarkable except for point tenderness along the lumbar (L4-L5) region of the spine. A complete blood count showed no leukocytosis and plain radiography of the lumbosacral spine showed a Schmorl’s node in the inferior endplate of L5. The patient was diagnosed with a trauma-induced Schmorl’s node and was treated with physical therapy, ice packs, and non-steroidal anti-inflammatory drugs. Her symptoms improved over the next several months. For patients with a history of axial load trauma and persistent back pain, clinicians should consider the possibility of a trauma-induced Schmorl’s node. Plain radiography or magnetic resonance imaging can help with the diagnosis and guide further management.

## Introduction

Low back pain is one of the most common presenting health problems in the primary care, outpatient setting with a one-year prevalence estimated to be 22–65% [1]. Most adults will present to their doctor with a complaint of low back pain at some point in their lives [2]. While most cases improve rapidly within the first few weeks, up to 20% of patients will have persistent pain lasting more than one year with substantial limitations in activity [3]. Even though the differential diagnosis for low back pain is broad, non-specific musculoskeletal pain accounts for the vast majority of cases in both adults and children [2-3]. This can be quite frustrating for both patients and physicians since management of non-specific back pain can be a slow and difficult process. One specific etiology of low back pain is the Schmorl’s node (SNs) which is a vertebral lesion first described by Christian Georg Schmorl in 1927 wherein the nucleus pulposus of an intervertebral disk herniates vertically through the endplate of an adjacent cranial or caudal vertebra [4]. The clinical significance of SNs is still in debate, given that they appear in many asymptomatic individuals; however, several studies have shown a high association of SNs with degenerative disk disease and lower back pain [4-5]. Informed consent statement was obtained for this study.

## Case presentation

An 18-year-old Native American female with a past medical history significant for a bicycle versus motor vehicle accident presented to urgent care for evaluation of low back pain. The patient reported that she had waxing and waning low back pain ever since her bicycle collision four months prior, but for the last six weeks, it had insidiously increased to an unbearable intensity. On presentation, she described her pain as a dull, throbbing pain lasting all day, which was localized to the lower back region without radiation elsewhere. She rated her pain as “10/10” and noted that it worsened with prolonged immobility; however, the pain was not exacerbated by movement. She was previously treated with acetaminophen, ibuprofen, hydrocodone, and tramadol with little effect. The patient did not have any recent sick contacts or travels, and she did not believe her pain was related to her bicycle accident. She denied fever, chills, night sweats, weight loss, bladder and bowel incontinence, weakness, numbness, and saddle anesthesia. Except for obesity and her previously mentioned bicycle accident, her medical history was otherwise unremarkable. The patient was evaluated in the emergency department (ED) at the time of her accident and reported that she sustained no fractures or significant soft tissue injury. She had no prior surgeries, and her family history was also unremarkable. She was a student at a local community college and lived at home with her parents, boyfriend, and 16-month old son. The patient denied smoking or illicit drug use, but she endorsed the occasional alcohol use.

On presentation, her vital signs were as follows: blood pressure; 124/76 mmHg, heart rate; 84, respiratory rate; 16, temperature (oral), 36.6°C, and oxygen saturation (room air), 97%. Her body mass index (BMI) was 38.0. On physical examination, she was obese, alert and oriented, and in obvious pain. She had no gait abnormalities. There were no visible lesions, rashes, ulcers, or ecchymosis throughout her body, and there was no visible evidence of scoliosis, kyphosis, or lordosis. There was midline point tenderness along the spine in the L4-L5 region without significant para spinal muscle tenderness. No pulsatile masses were appreciated on the abdominal exam, and there was no costovertebral angle tenderness. The patient was completely neurovascularly intact and had full strength in all major muscle groups throughout her bilateral upper and lower extremities. All deep tendon reflexes were 2+ and symmetric, and the Babinski reflex was absent bilaterally. Forward flexion and extension of the spine were limited due to pain, but there was a full, active range of motion with lateral flexion. Straight leg raise was negative bilaterally. The remainder of her physical exam was unremarkable.

**Figure 1:**
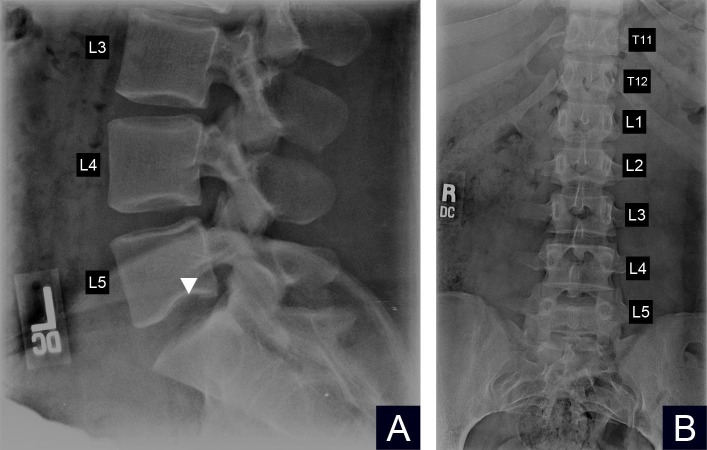
Figure showing the plain radiographs of the lumbar spine A) Lateral view of the lower lumbar spine showing a small Schmorl’s node in the inferior endplate of lumbar spine (L5) (white arrowhead) B) The anteroposterior view of the lumbar spine showing unremarkable findings

A complete blood count with differential showed no leukocytosis or other abnormalities. Because of point tenderness along the patient’s lower lumbar spine, lumbar radiographs were obtained. These radiographs showed normal bone density, normal alignment, and curvature of the spine, no evidence of acute fractures and overall no significant degenerative changes. However, there was a small Schmorl’s node noted in the inferior endplate of L5 best seen on the lateral lumbar view (Figure 1A). The anteroposterior (AP) view showed unremarkable findings (Figure 1B). Given the history of previous trauma, lower lumbar point tenderness on physical exam and radiographic findings consistent with an SNs, the patient was diagnosed with low back pain secondary to a trauma-induced SNs. The patient was then referred to an orthopedic spine surgeon who recommended physical therapy, weight loss, ice packs, and non-steroidal anti-inflammatory drugs (NSAIDs) as initial management. She was informed that surgical treatment existed if her symptoms persisted despite conservative management. The patient agreed with conservative management, and her pain improved over the next several months.

## Discussion

Low back pain can be challenging to manage given the broad spectrum of underlying etiologies, including infectious, inflammatory, neoplastic, autoimmune, musculoskeletal, visceral, and psychosocial causes [2, 6]. Even though the majority of low back pain can be attributed to non-specific musculoskeletal pain [2- 3], it is still important for clinicians to evaluate for more serious underlying causes of low back pain. Having a systematic approach in the evaluation of low back pain is critical for diagnostic accuracy and subsequent management.

Work-up for our patient began with a thorough history and physical exam which was mostly unremarkable except for a history of trauma and point tenderness along the lower lumbar spine. The patient was previously told that she had a nonspecific musculoskeletal strain from her traumatic event, but on physical exam, there was no diffuse para spinal tenderness. This prompted further investigation for an alternative diagnosis. Evidence suggests that obtaining plain radiography in patients with non-specific low back pain does not improve outcomes or change management, and it should be avoided if possible in young patients to limit unnecessary gonadal radiation [3]. However, there are certain red flags in the patient’s history and physical exam that may justify obtaining plain imaging such as constitutional symptoms (fevers, night sweats, or unexplained weight loss), focal neurological deficits, prolonged corticosteroid use, or a history of malignancy or trauma [3, 6]. The decision was therefore made to obtain plain imaging since our patient had spinal point tenderness and a history of trauma with no significant improvement of pain despite conservative management over a period of four months. Plain radiography revealed a Schmorl’s node in the inferior endplate of L5 which led to the diagnosis of low back pain secondary to a trauma-induced SNs.

Intervertebral disk herniation typically occurs horizontally, leading to impingement of the spinal cord which can potentially cause radicular symptoms or other spinal cord pathologies; however, disk herniations can also occur vertically and extend through the endplate of an adjacent cranial or caudal vertebra: a phenomenon known as a Schmorl’s node. Although the exact etiology of SNs is unknown, there are several proposed pathophysiological mechanisms to explain their development, including increased axial stress on the vertebral endplates at weak points, disk degeneration, embryological defects, and autoimmune processes [4-5]. The latter three mechanisms may predispose patients to SNs formation and may be important in potentiating SNs development when other risk factors are present [4]. Unlike the other mechanisms, however, increased axial load on the vertebral endplate due to trauma can independently lead to the SNs formation, as evidenced by studies showing the statistically significant increased prevalence of SNs in motorcyclists involved in collisions [4]. Most epidemiological studies show an increasing prevalence of SNs with age, and the majority of cases occur past the fourth decade of life with very few cases in patients younger than an age of 20 years [4, 7-8]. Most SNs are found in the thoracolumbar thoracic vertebrae (T8-L1) region of the spine [ 4-5, 7]. Diagnosis can be made with plain radiography, however acute traumatic SNs might not be adequately visualized immediately after the traumatic event [9]. Additionally, SNs may not be visible on anteroposterior views as was the case with our patient. Magnetic resonance imaging (MRI) is the gold standard diagnostic approach due to its ability to differentiate between symptomatic and asymptomatic SNs by visualizing edema in T2-weighted images with simultaneous low signal intensity on T1-weighted images [4-5]. Cadaver-based studies have revealed a high prevalence of asymptomatic SNs in the general population, but symptomatic SNs tend to be extremely painful with a significant decline in quality of life [4, 10]. Other than typical findings on MRI, it is still unclear as to why certain SNs are painful while others are asymptomatic.

Clinical suspicion for an SNs in our patient was low given her young age and uncharacteristic location in the lower lumbar spine; however, given her history of axial load trauma and symptoms that did not improve with conservative management, a diagnosis other than non-specific low back pain was considered. Even though the initial treatment for SNs is the same as non-specific musculoskeletal pain (conservative management with NSAIDs), it is important to make the diagnosis of an SNs, if one exists, so that further treatment options can be pursued, including segmental fusion surgery, percutaneous fluoroscopy-assisted vertebroplasty, tumor necrosis factor-alpha inhibition, or ramus communicans nerve block [4-5]. MRI can be useful in determining which SNs is symptomatic and therefore which patients may benefit from treatment with these more invasive methods.

## Conclusions

Clinicians should be aware that not all low back pain is a non-specific musculoskeletal pain. If a patient with a history of axial load trauma presents with persistent low back pain and unremarkable imaging at the time of trauma, consider the possibility of a Schmorl’s node. Plain radiography of the thoracolumbar spine may be sufficient for the diagnosis, but MRI is more sensitive and can help guide further treatment since it is able to differentiate between symptomatic and asymptomatic SNs.
